# A Comparative Study of Propofol and Isoflurane Anaesthesia using Butorphanol in Neurosurgery

**Published:** 2009-06

**Authors:** LD Mishra, N Rajkumar, SN Singh, RK Dubey, G Yadav

**Affiliations:** 1 Professor and Head, Division of Neuroanaesthesia; 2,3 P.G.Student, Department of Anaesthesiology, Institute of Medical Sciences, Bananas Hindu, University, Varanasi-221 005; 4,5 Lecturer, Department of Anaesthesiology, Institute of Medical Sciences, Bananas Hindu, University, Varanasi-221 005

**Keywords:** Craniotomy, Propofol, Isoflurane, Butorphanol, Haemodynamics, Extubation time, Recovery of consciousness

## Abstract

**Summary:**

Propofol and isoflurane have well proven roles as intravenous and inhalational anaesthetics respectively in neurosurgery. We conducted this study to know the outcome using butorphanol as an intraoperative analgesic. Sixty craniotomy patients randomly divided into two groups of 30 each were included in this study. Group A patients were induced and maintained with propofol. Group B patients were induced with thiopentone and maintained with isoflurane. All patients were administered 30μg.kg^−1^ butorphanol intravenously 10 minutes before induction of anaesthesia, followed by slow injection of 30μg.kg^−1^ midazolam. All were assessed for sedation, respiratory insufficiency, postoperative nausea and vomiting (PONV) and other side effects in the recovery room. We found no difference in demographic parameters between the groups. The fall in HR was maintained in the post induction / intubation period and throughout the intraoperative period in Group A, unlike Group B patients in whom it rose significantly following intubation. Butorphanol was found to be a safe intraoperative analgesic in neurosurgical patients. In addition, it was associated with statistically better haemodynamics and earlier recovery when used with propofol as compared to thiopentone-isoflurane anaesthesia.

## Introduction

Propofol and isoflurane have well proven roles as intravenous and inhalational anaesthetics respectively in neurosurgery^1^. As most of the neurosurgical procedures are of longer duration, it is quite reasonable that we use a relatively longer acting analgesic which can give an equianalgesic intra operative period than a shorter acting newer opioid which needs to be repeated frequently or given by continuous infusion. On the other hand, early neurological assessment is essential following most neurosurgical operations. Thus we need to use drugs and techniques that should not cause any hindrance to this objective. Butorphanol has been reported to provide adequate analgesia when used as a supplement in balanced anaesthetic techniques^2^. In healthy volunteers, butorphanol (0.03-0.06mg.kg^−1^ IV) produces no or minimal cardiovascular changes^3^.

Medline search did not reveal any information regarding intraoperative conditions and patient outcomes in neurosurgical patients with butorphanol/propofol vs butorphanol/thiopentone/isoflurane. Accordingly, we planned this study to evaluate the intraoperative conditions and patient outcomes in neurosurgical patients using butorphanol/propofol Vs butorphanol/thiopentone/isoflurane anaesthesia.

## Methods

The study was conducted at the SS University Hospital, Institute of Medical Sciences, Banaras Hindu University, Varanasi. After obtaining the institutional ethical committee approval and informed consent, sixty patients of ASA grade I/II with Glasgow Coma Scale Score of 13 or more, posted for elective craniotomy were included in the study. The patients were randomly assigned into two groups A and B of thirty patients each.

Group A patients were induced and maintained with propofol. Group B patients were induced with thiopentone and maintained with isoflurane.

**Exclusion Criteria:** Patients with systemic disorders like hypertension, diabetes mellitus, respiratory diseases, hepatic or renal insufficiency were excluded from the study. Patients with history of allergy to any drug used in the past were also excluded from the study.

**Study Procedure:** All patients were premedicated with oral alprazolam 0.25-0.5mg at evening and 6.00 AM on the day of surgery. Baseline heart rate (HR), mean arterial blood pressure (MABP), oxygen saturation (SpO_2_), body temperature and central venous pressure (CVP) were recorded. 10 minutes before induction of general anaesthesia (GA) 30mcg.kg^−1^ butorphanol followed by slow injection of 30mcg.kg^−1^ of midazolam were administered intravenously (IV). Group A patients were induced with propofol bolus titrated to the disappearance of verbal response and Group B patients were induced with thiopentone titrated to the loss of eyelash reflex. All patients were intubated with a flexometallic tube of appropriate size 3 min after giving 0.1 mg.kg^−1^ of vecuronium bromide. Care was taken to prevent injuries to eyes, ears, peripheral nerves or limbs due to positioning.

After induction the anaesthesia was maintained with propofol and nitrous oxide in oxygen (60:40) in Group A patients. Propofol infusion was given in a dose range of 50-150 mcg.kg^−1^.min^−1^, titrated to the haemodynamic parameters. In Group B patients, the anaesthesia was maintained with isoflurane and nitrous oxide in oxygen (60:40). The end tidal concentration of isoflurane was titrated to keep the haemodynamic parameters near to base line values. Intermittent doses of vecuronium bromide were given in both the groups as and when required. The central venous pressure& end tidal carbon dioxide (EtCO_2_) were maintained in the range of 7-10 cm of water & 30-35 mmHg respectively in both the groups. The anaesthetic was stopped after skull pin site closure in all patients. The HR, MABP, SpO_2_, EtCO_2_, esophageal temperature, anaesthetic gas concentrations and urine output were monitored in all patients.

We extubated all patients on the operating table after recovery of adequate spontaneous ventilation and shifted them to the recovery room after recovery of consciousness. Patients in whom extubation was delayed and/or needed elective ventilation were noted. The time interval between cessation of the anaesthetic agent, extubation and recovery of consciousness were recorded. In the recovery room the patients were assessed for sedation, respiratory insufficiency, postoperative nausea and vomiting (PONV) and other side effects, if any. We used Ramsay sedation score for the assessment of sedation (Score1= Anxious, agitated, non-cooperative; Score 2= Cooperative, oriented, tranquill; Score 3= Respond to verbal commands; Score 4= Brisk response to loud noise or a light tap; Score 5= Sluggish response to loud noise or a light tap; Score 6= No response to stimuli). Unpaired t test was used for statistical analysis and *p*<0.05 was considered as significant.

## Results

We did not find any difference between the groups in terms of demographic parameters and duration of anaesthesia (Table 1).

**Table 1:** Demographic Data(Mean±SD)

Variable	Group A(n=30)	Group B(n=30)	T value	p-value*
Age(years)	39.00±13.08	39.20±12.44	−0.043	0.966
Weight (Kg)	57.33±4.43	57.33±4.78	0.000	1.000
Duration of anaesthesia(min)	171.47±27.57	169.47±26.45	0.203	0.841

* p-values less than 0.05 were taken as significant

There was a significant fall in HR following midazolam and butorphanol in both groups. The fall in HR was maintained in the post induction / intubation period and throughout the maintenance of anaesthesia in Group A, but not in Group B patients in whom it rose significantly following intubation. The HR was not significantly different from the baseline throughout the maintenance of anaesthesia in either group at most of the intervals (Table 2).

**Table 2:** Inter group comparison of heart rates (beats per min)(Mean ± SD)

Time Interval	Group A(Mean ± SD)	Group B(Mean ± SD)	t-value	p-value
1.(Base line)	86±3.14	83.00±3.14	1.88	0.07
2.(Post midazolam)	82±3.14	80.00±3.14	1.75	0.09
3.(Post induction)	76±3.14	82.00±3.14	−5.23	0.00*
4.(Post intubation)	82.20±3.17	90.00±3.14	−6.79	0.00*
5.(Post extubation)	88±3.14	92.00±3.14	−3.49	0.00*
6.(Post recovery of consciousness)	84±3.14	86.00±3.14	−1.75	0.09

* p-values less than 0.05 were taken as significant

**Fig 1:**
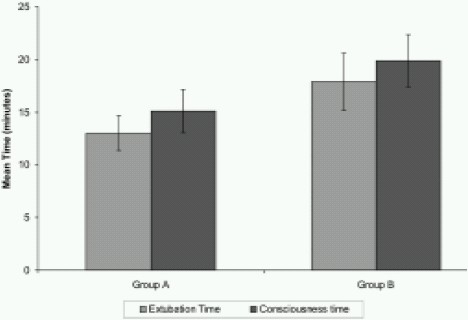
Mean extubation and recovery of consciousness time of the two groups

Table 3 shows the intergroup comparison of MABP at different intervals. The baseline and post midazolam/ butorphanol value were not significantly different. All the values in the rest of the periods were significantly higher in Group B patients when compared to Group A. An intergroup comparison of mean extubation time and time to recovery of consciousness after the cessation of anaesthetic is shown in Fig 1. The mean extubation and recovery of consciousness time in Group A patients were 13.00 ± 1.65 minutes and 15.07 ± 2.05 minutes respectively as compared to 17.67 ± 2.70 and 19.87 ± 2.45 minutes respectively in Group B. It is evident that both the times are significantly longer in Group B.(p=0.000)

**Table 3:** Inter group comparison of mean arterial blood pressure(mmHg) at different intervals (Mean ± SD)

Time Interval	Group A	Group B	t-value	p-value
1 (Base line)	82.00±3.14	84.00±3.14	−1.75	0.09
2 (Post midazolam)	80.00±3.14	82.00±3.14	−1.87	0.07
3 (Post induction)	72.00±3.14	78.53±4.05	−4.94	0.00*
4 (Post intubation)	76.00±3.14	88.53±4.05	−9.47	0.00*
5 (Post extubation)	84.67±2.77	87.27±3.52	−2.16	0.04*
6 (Post recovery of consciousness)	82.00±3.14	84.93±2.99	−2.40	0.02*

* p-values less than 0.05 were taken as significant

**Fig 2:**
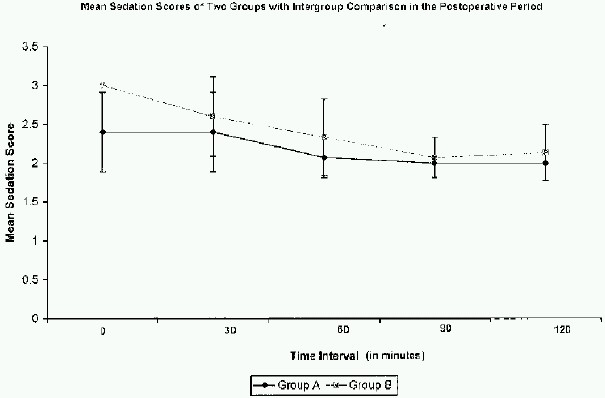
Mean sedation scores of two groups with intergroup comparison in the postoperative period

The mean sedation score was significantly higher in Group B patients when compared with Group A at the time of admission to recovery room. The sedation scores at other time intervals were not significantly different between the two groups (Fig 2).(p=0.000)

One patient in Group B developed bronchospasm following extubation. None of the patients needed admission to the intensive care unit.

## Discussion

Unfortunately there are very few studies reported in the literature on butorphanol and its influence on intraoperative conditions and patient outcomes. More specifically there is no report on the use of butorphanol in neurosurgical patients. Pandit and Kothary,^4^ observed that thiopentone/ butorphanol induction provides statistically insignificant haemodynamic responses to tracheal intubation in laparoscopic outpatient procedures. Laffey and Kay^5^ in a comparative study on butorphanol and morphine as a premedicant found that butorphanol was as effective as morphine with the advantage of fewer unwanted side effects.

Yung-Fong Sung et al^6^ found that the butorphanol was a good opioid analgesic for balanced anaesthesia. The authors suggested that butorphanol was a better choice than morphine for use in balanced anaesthesia techniques because of its comparable analgesic efficacy and amnesia along with lesser postoperative respiratory depression and a shorter recovery room stay. Pramila Chari et al^7^ observed conducive LMA insertion conditions with the use butorphanol as compared to fentanyl. Anil Agarwal et al^8^ observed the pain relieving property of butorphanol premedication given prior to intravenous propofol. This pain relieving property has a favourable effect prior to neurosurgical procedures by lowering patient's anxiety and the accompanying hemodynamic alteration.

Midazolam acts synergistically with general anaesthetics. McClunes et al al^9^ observed a synergistic interaction of midazolam with propofol to loss of response to verbal commands as the clinical end point. Oliver et al^10^, observed that midazolam premedication reduces propofol dose requirements for multiple anaesthetic end points.

In our study, we found that administration of midazolam and butorphanol 5 minutes before induction of GA produced a statistically significant fall in HR and MABP in both the propofol and isoflurane groups. We may attribute this to anxiolysis and synergistic sedative effects of midazolam and butorphanol. The fall in HR & MABP was not significant when compared between the two groups which shows a consistency of effect.

Grounds et al^11^ found no change in heart rate following injection of propofol whereas there was tendency to initial tachycardia following thiopentone. Coley et al^12^ observed that propofol attenuates the increase in arterial blood pressure and heart rate in response to laryngoscopy and intubation. They also reported that this increase in arterial pressure was associated with an increase in plasma noradrenalin levels after thiopentone induction level but not after propofol. We observed an increase in mean heart rate and MABP after tracheal intubation in Group B patients but the rise was not significant when compared to baseline. This suggests that the propofol maintains the baseline hemodynamics. On comparing the haemodynamics between the groups we found that the HR and MABP were always on the higher side following intubation, during maintenance of and emergence from anaesthesia in patients anaesthetised with thiopentone/ isoflurane anaesthesia.

Todd et al^13^, in their prospective comparative trial of three anaesthetics for elective supratentorial craniotomy reported higher heart rates in isoflurane/nitrous oxide anaesthesia. Van Hamelrijck et al^14^, in their study on craniotomy patients using thiopentone sodium/ isoflurane and fentanyl/ nitrous oxide anaesthesia, observed that the decrease in MABP after induction with thiopentone was followed by a significant increase in MABP and HR during intubation. Conversely the HR and MABP did not change during propofol loading infusion. Our study observation is in accordance with these studies.

Billard et al^15^, reported a significant increase in mean blood pressure (mean 50 mmHg, p<0.05) following intubation and that the haemodynamic response to intubation was decreased by the administration of fentanyl in a dose dependent manner. Our observations are similar to the study of Billard et al, despite the fentanyl being replaced by butorphanol in our study. Thus we may say that butorphanol can also blunt the haemodynamic response when used with propofol, again an advantageous factor in procedures related to neurosurgery.

The mean extubation and recovery of consciousness time in Group A patients were 13.00 ± 1.65 minutes and 15.07 ± 2.05 minutes respectively as compared to 17.67 ± 2.70 and 19.87 ± 2.45 minutes respectively in Group B. This goes in line with similar studies of Ebert et al^16^, and Alan et al^17^, where time to recovery was found to increase with increasing duration of isoflurane anaesthesia but not after propofol anaesthesia. Alan et al^17^ reported a mean emergence and extubation times of 20.8 ± 10.1 and 30.0 ± 28.0 minutes respectively in their observations. Valance^18^ also reported prolonged recovery and psychomotor impairment with isoflurane anaesthesia.

One patient from Group B had bronchospasm immediately after extubation which could have been due to the presence of traces of isoflurane in the breathing circuit. Though this was found in only one patient, it cannot be ignored altogether seeing a relatively small number of patients in the study groups. None of our patients had any other adverse effects such as emergence agitation or PONV in the recovery period.

The clinician has to be aware of issues regarding the context sensitive half life of fentanyl and sufentanil, which being relatively short will require frequent topup doses or infusions and fentanyl and sufentanil are more costaly as compared to butorphanol, Thus we may conclude that butorphanol can also be used for intraoperative analgesia in neurosurgical operations. It is associated with statistically better haemodynamics and earlier recovery when used with propofol as compared to thiopentone-isoflurane anaesthesia.
